# Multiple Myeloma With a Rare Presentation at Preserved Uninvolved Immunoglobulins

**DOI:** 10.7759/cureus.25513

**Published:** 2022-05-31

**Authors:** Shunsuke Soma

**Affiliations:** 1 Department of General Practice, Emergency and Critical Care Center, Aomori Prefectural Central Hospital, Aomori, JPN

**Keywords:** quantitation of immunoglobulins, uninvolved immunoglobulins for symptomatic myeloma, monoclonal gammopathy of undetermined significance, m protein, monoclonal immunoglobulins, delayed diagnosis, fever of unkown, immunoglobulin d multiple myeloma, diagnosis of multiple myeloma

## Abstract

Multiple myeloma is a neoplastic disease of plasma cells that produces monoclonal immunoglobulins, known as monoclonal proteins, causing hypercalcemia, anemia, renal insufficiency, bone lesion, and other organ damage. In most multiple myeloma, the amount of monoclonal protein correlates with the tumor burden and reflects its prognosis. Uninvolved immunoglobulins are often suppressed as monoclonal protein levels increase as a result of the increasing percentage of myeloma cells in the bone marrow. Detection of monoclonal protein by protein electrophoresis, immunofixation, and serum-free light chain assay is a highly sensitive analysis and is not suitable as a screening test because it is positive in a few percent of healthy elderly people, the majority of whom are classified as benign monoclonal gammopathy of undetermined significance. Therefore, it is widely used in practice to measure quantitation of immunoglobulins by nephelometer, and only when uninvolved immunoglobulins are decreasing in the values for each subtype, monoclonal protein identification by immunofixation, serum-free light chain assay, and bone marrow examination must be additionally performed for proactively diagnosing multiple myeloma. However, we experienced symptomatic multiple myeloma in which uninvolved immunoglobulins were not suppressed and there were no significant changes in immunoglobulin levels despite the relatively rapid progression. It would suggest that if you suspect symptomatic myeloma, you should not rule out the possibility of multiple myeloma because of preserved uninvolved immunoglobulins in laboratory findings.

## Introduction

Multiple myeloma is a neoplastic lesion caused by mature immunoglobulin-producing plasma cells and is characterized by the presentation of hypercalcemia, renal dysfunction, anemia, and bone lesions (CRAB features) as organ damage features. Normal plasma cells produce various subtypes of immunoglobulins such as IgG, IgM, and IgA, while myeloma cells are characterized by the production of monoclonal immunoglobulin (M-protein). Furthermore, as myeloma proliferates in the bone marrow, normal plasma cells are reduced, and the production of uninvolved immunoglobulins other than M-protein is suppressed. As a result, multiple myeloma is associated with immune paresis and a high risk of bacterial and viral infections [1]. Protein electrophoresis (SPEP), immunofixation (IFE), and free light chain assay (FLC) of the serum are very sensitive and almost all are diagnosed as monoclonal gammopathy of undetermined significance (MGUS), although it is found in a few percent of patients over 50 years of age when performed as a screening test [2-4]. Of these, approximately 0.5-1% per year would progress to multiple myeloma in the future, and even over a 30-year course, only about 30% would develop multiple myeloma [5-7]. This means that the majority of patients diagnosed with MGUS could be likely to die before progressing to myeloma and requiring treatment. Because SPEP, IFE, and FLC as screening tests could be overdiagnosed, these tests are only performed in cases of strongly suspected symptomatic multiple myeloma. However, a symptom such as the CRAB feature seen in multiple myeloma is very common in the elderly and is more often caused by diseases other than myeloma. Therefore, even when M-protein is detected, the question arises as to whether it is due to multiple myeloma. Therefore, quantitative immunoglobulin assay by nephelometer is often used to estimate the presence of tumor cell proliferation in the bone marrow based on whether the production of normal immunoglobulins other than M-protein is suppressed. A previous report showed that normal immunoglobulins were suppressed in 90% of multiple myeloma cases [8], but only 30% of MGUS [5]. It can also be inferred from the fact that hypergammaglobulinemia due to non-neoplastic diseases seen in chronic inflammatory diseases, liver cirrhosis, and nephrotic syndrome is elevated in all isotypes. However, we report here a case of hypercalcemia and pain associated with bone lesions in a patient with clear symptomatic myeloma who did not show normal immunoglobulin suppression and required a longer time to be diagnosed.

## Case presentation

**Figure 1:**
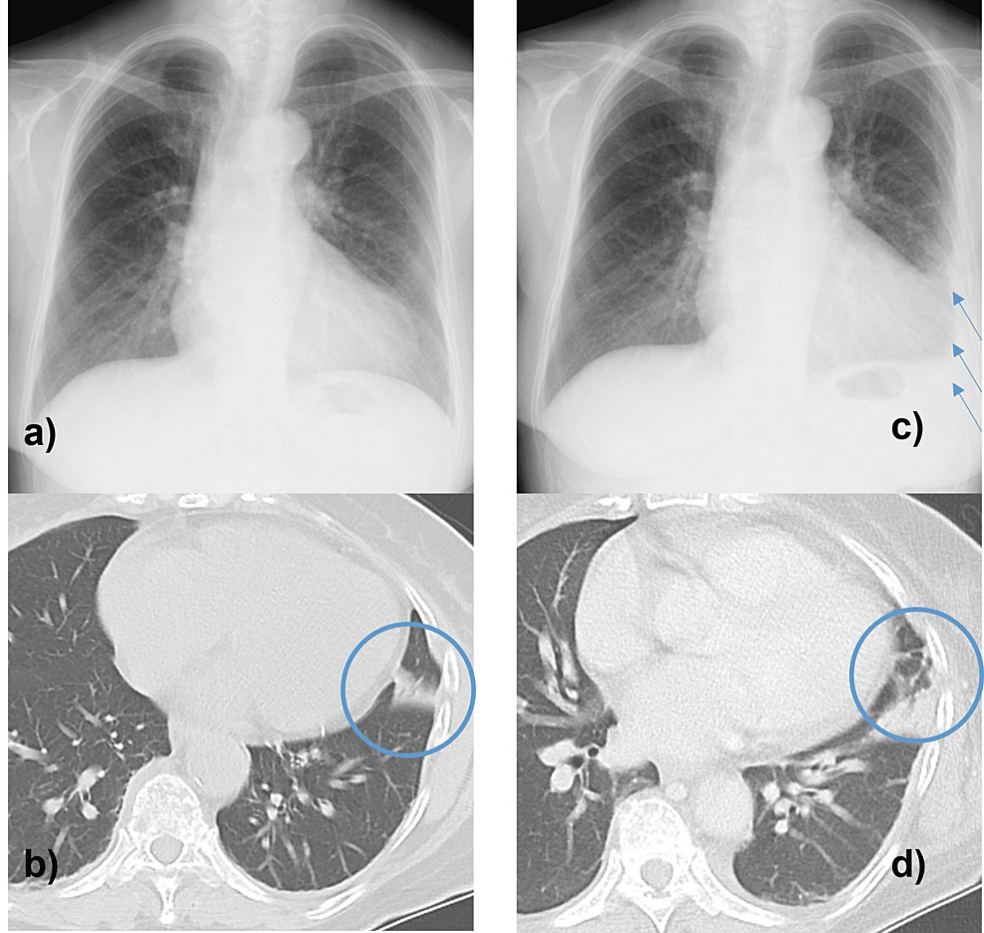
Chest X-ray and CT showing progressive infiltration shadow in S8 left lung and increasing pleural effusion a) Chest X-ray at the initial visit, b) Chest CT at the initial visit, c) Chest X-ray after 2 weeks, d) Chest CT after 2 weeks

A 67-year-old woman had a slight fever of 37°C and chest pain on the left side for a month. Her doctor prescribed painkillers and vitamins for intercostal neuralgia. She had persistent pain and a fever over 38°C for a week, so she visited our emergency room on Sunday. Computed tomography (CT) showed a slight infiltration shadow in S8’s left lung and oral antimicrobial agents were started with the diagnosis of left pneumonia and pleurisy. Another week later, in addition to fever, hypercalcemia was noted with serum-corrected calcium of 12.7 mg/dl. The patient had pleurisy refractory to antimicrobial agents, and hypercalcemia due to pleural involvement by tuberculous pleuritis or neoplastic disease was suspected. Because hypercalcemia required correction for infusion, the patient was admitted to the hospital for a thorough examination. Tuberculous pleurisy was ruled out due to a negative Interferon Gamma Release Assay. In addition to hypercalcemia, multiple myeloma was also suspected because of mild renal impairment and anemia. However, the serum immunoglobulin IgG subtype was slightly increased, and other subtypes (IgM, IgA) were not suppressed. As a result, we ruled out the possibility of myeloma at this point. CT and chest X-ray was performed again, which showed progressive infiltration shadow and increased pleural effusion (Figure 1), leading to a strong suspicion of malignant lung or pleural disease.

**Figure 2:**
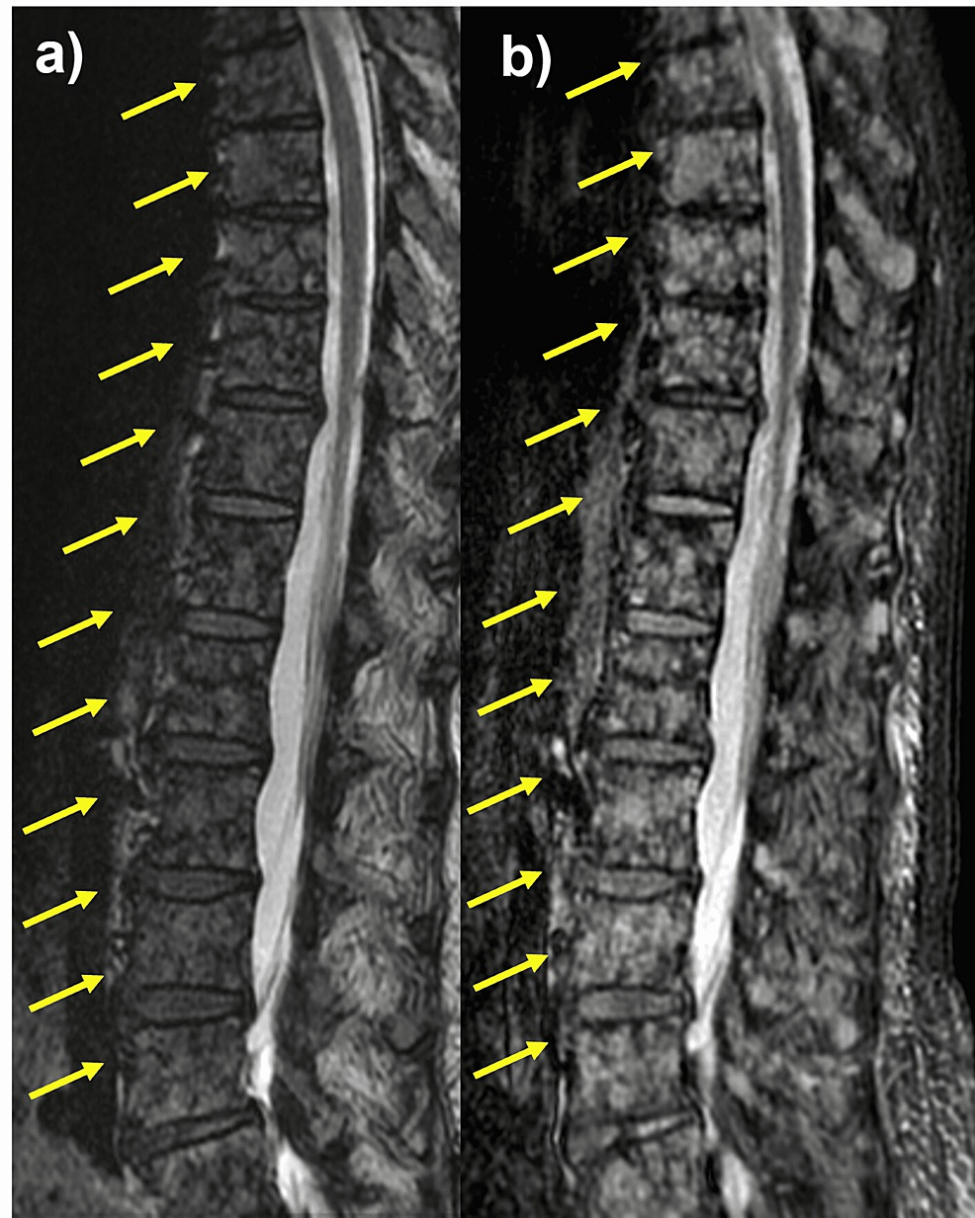
A lumbar spine MRI showing mottled high signal in the lumbar vertebral body a) MRI T2 emphasized image, b) MRI STIR image STIR: Short inversion time inversion-recovery

We consulted respiratory medicine. Bronchoscopy was performed for left S8’s lesion but found only nonspecific inflammatory findings. About a month and a half after the initial visit, lumbar back pain appeared again, and a lumbar magnetic resonance imaging (MRI) was performed, which showed a high-signal pattern in the lumbar vertebral body at T2 emphasized image and short inversion time inversion recovery (STIR) image (Figure 2).

On the other hand, there was no evidence of bone destruction, and a hematopoietic tumor was suspected rather than bone metastasis of cancer. The hypercalcemia and pain associated with the bone lesions suggested the possibility of multiple myeloma, and immunoglobulin measurements revealed only a mild increase in IgG and no suppression of other immunoglobulins. Quantitation of immunoglobulins is seen in Table 1. 

**Table 1:** Quantitation of immunoglobulins by nephelometer (1) Serum immunoglobulin levels at 2 weeks after the first visit, (2) Serum immunoglobulin levels at one and a half months after the first visit

	(1)	(2)	Normal range
TP g/dl	7.6	7.2	6.6-8.1
Alb g/dl	2.7	2.2	4.1-5.1
IgA mg/dl	157.7	120.3	93.0-393.0
IgG mg/dl	2715	2838.1	861.0-1747.0
IgM mg/dl	58.8	48.9	50.0-269.0

**Figure 3:**
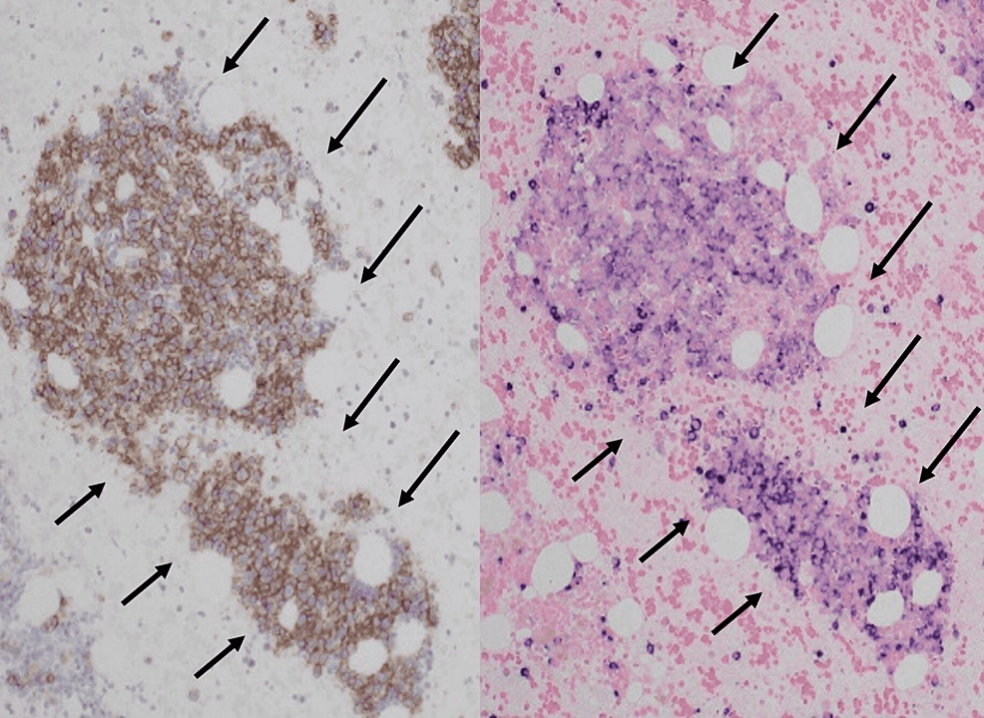
Bone marrow specimen (X100) Bone marrow specimens showing CD38 positive (left side) and κ-ISH positive (right side) cells increasing monotonously

However, serum protein fractionation on cellulose membranes suggested the presence of M-protein, and immunofixation revealed IgG-κ-type M-protein. A bone marrow examination performed by the hematologist showed a remarkable increase in monotonous plasma cells (Figure 3).

**Figure 4:**
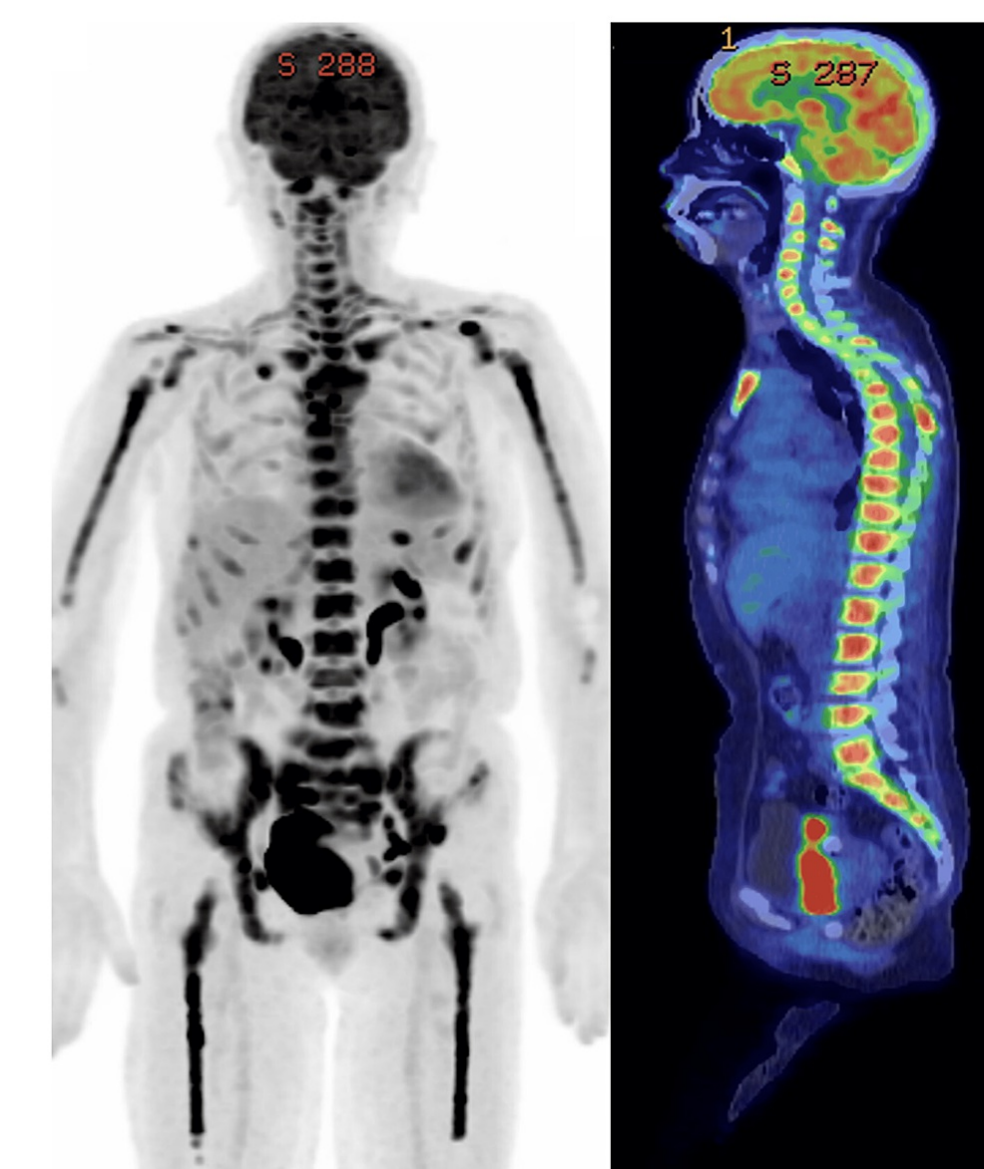
PET-CT showing diffuse fluorodeoxyglucose accumulation in almost all bones in the body PET-CT: Positron emission tomography-computed tomography

Positron emission tomography-computed tomography (PET-CT) showed diffuse accumulation in the bones throughout the body (Figure 4).

She was finally diagnosed with symptomatic multiple myeloma. She was treated with BD regimen (bortezomib 1.3mg/m^2^, day 1, 4, 8, 11 dexamethasone 20mg/body, day 1, 4, 8, 11) and DBD regimen (daratumumab 16mg/kg, day 1, 8, 15; bortezomib 1.3mg/m^2^; day 1, 4, 8, 11 dexamethasone 20mg/body, day 1, 2, 4, 5, 8, 9, 11, 12) at the Department of Hematology, but myeloma progressed. She was switched to an ELD regimen (elotuzumab 10mg/kg, day 1, 8, 15, 22, dexamethasone 28mg/kg, day 1, 8, 15, 22) and achieved a very good partial response, but complications of severe infection in the second cycle required hospitalization. After that, the patient was unable to continue treatment due to a decline in performance status, and only palliative care using steroids alone was provided. She died approximately six and a half months after our first visit for myeloma progression.

## Discussion

This patient presented with a fever of unknown origin that persisted for more than three months and was eventually diagnosed with multiple myeloma. It took about two months from the initial diagnosis to the definitive diagnosis, making this a difficult case to diagnose. The patient had left-sided chest pain as an accompanying symptom and a very small amount of pleural effusion on X-ray and CT, which may have contributed to our recommendation to focus on pleural disease for differentiation. During the disease, the patient developed hypercalcemia, and multiple myeloma was suggested as a possible diagnosis. However, since IgG was only mildly elevated in the serum immunoglobulin quantitative test and other immunoglobulins such as IgA and IgM were not suppressed, we judged that the patient had polyclonal hypergammaglobulinemia associated with chronic inflammation and did not search for clonality by SPEP, IFE and FLC assay. Anemia was also present since the initial visit, but since it was thought to be a fever of unknown origin, it was judged to be anemia associated with chronic inflammation. Iron kinetic tests also showed decreased iron (Fe) and total iron-binding capacity (TIBC) and increased ferritin, which was consistent with anemia associated with chronic inflammation. Two of the CRAB features for multiple myeloma were applicable, but the absence of normal immunoglobulin suppression ruled out myeloma altogether, which contributed to the delay in diagnosis.

The diagnosis of multiple myeloma is generally based on three factors: (1) the presence of M-protein; (2) an increase in plasma cells in the bone marrow; and (3) the presence of organ damage such as hypercalcemia, anemia, renal damage, and bone lesions. Generally, M-proteins are identified by SPEP, IFE, and FLC assay. But these tests are too sensitive, and when performed as screening tests, they are often suspect-positive. The majority of these patients have MGUS with no increase in tumor cells in the bone marrow and no organ damage. Kyle et al. found that 3.2% of patients over 50 years of age were diagnosed with MGUS when SPEP was performed on residual blood serum collected at the Mayo Clinic as part of routine laboratory testing. The rate increases with age, from 5.3% in patients aged 70 years and older to 7.5% in patients aged 85 years and older [2]. Furthermore, Landgren et al. reported that in the general population, the prevalence of MGUS is 2.4% in those over 50 years of age, and by race, it is more common in blacks than in Hispanics or whites [4]. Although 0.5-1% of patients develop multiple myeloma each year, with a 30-year risk of progression [5-7], only a small percentage of patients are eligible for treatment, and many cases require follow-up for decades, which is excessive in terms of patient and healthcare professional burden and cost. It is essential to diagnose multiple myeloma requiring treatment at the appropriate time and without delay. We have used quantitation of immunoglobulins by nephelometer as a screening test. If some or all immunoglobulin subtypes are suppressed, symptomatic myeloma is strongly suspected. Kyle et al. (2002) analyzed the characteristics of 1027 patients with newly diagnosed multiple myeloma and reported that 91% had one or more normal immunoglobulins suppressed [8]. On the other hand, MGUS followed up for 20~35 years reported that only 29% had suppressed normal immunoglobulins [5]. Quantitation of immunoglobulins by nephelometer is very convenient for screening because they can often be easily measured at one's facility and the results are immediately available in a short time. The overdiagnosis of MGUS can be reduced by performing quantitation of immunoglobulins by nephelometer and adding SPEP, IFE, and FLC assay only when uninvolved immunoglobulins are suppressed to aggressively search for and confirm the presence of M-proteins. However, as in this present case, uninvolved immunoglobulins are not suppressed in about 10% of symptomatic myeloma, so caution should be exercised when using this method for exclusion diagnosis. In our case, the patient had gradually increased back pain, and a lumbar MRI scan was performed to reexamine the possibility of myeloma. When symptoms are present that cannot be explained by other diseases, more sensitive IFE and FLC assay should be used in combination.

 The preservation of uninvolved immunoglobulin has been reported as a favorable prognostic factor in multiple myeloma. E Kastritis et al. compared 1755 patients with multiple myeloma whose serum immunoglobulins were measured in a Greek database with and without preserved uninvolved immunoglobulin [9]. Jennifer L. J. Heaney et al. showed similar results using a dataset of 5826 multiple myeloma patients in the United Kingdom [10]. It was reported that suppression of the IgM subtype is influenced by overall survival. Chuanying Geng et al. report 278 single-center multiple myeloma patients from China, but propensity score matching was performed and cytogenetic including R-ISS and other characteristics, which have also been shown to be independent prognostic factors [11]. A different immunoassay with nephelometer using the HLC® assay, which can measure subsets of immunoglobulin heavy and light chains, has also reported that the preservation of uninvolved immunoglobulins is a favorable prognostic factor associated with overall survival [12, 13]. Reports from a Danish registry and a single center in Turkey have not shown an association between the presence of suppression of uninvolved immunoglobulin and survival, but progression-free survival in the Danish report tended to be similar [14, 15]. In the present case, the patient had IgG-κ myeloma, but died rapidly about 6.5 months after diagnosis despite the levels of both IgM and IgA subtypes being within normal range; R-ISS corresponded to stage II, and no poor prognostic chromosomal abnormalities were detected by FISH. No poor prognostic factors other than age over 65 years and low PS 1~2 were found among the previously reported poor prognostic factors. The response to treatment with the bortezomib regimen was also poor. It may suggest that there are still unknown poor prognostic factors in our case.

## Conclusions

Screening for suspected symptomatic myeloma by quantitation of immunoglobulins is a simple and useful method to prevent overdiagnosis of MGUS and to identify symptomatic myeloma requiring treatment. It should be noted, however, that approximately 10% of multiple myeloma cases have preservation of uninvolved immunoglobulins. When symptoms such as CRAB feature are present and cannot be explained by other common diseases or pathologies, M-protein should be searched for by a more sensitive IFE or serum FLC assay. In addition, the preservation of uninvolved immunoglobulin in multiple myeloma at initial diagnosis has been reported as an independent favorable prognostic factor. However, in this case, the patient was refractory to treatment and had a poor prognosis. This patient’s clinical course could not be clearly explained by poor prognostic factors already reported. Therefore, we suppose that there may be unknown factors, and further case accumulation and investigation are needed.
